# Exosomal miR-1290 is a potential biomarker of high-grade serous ovarian carcinoma and can discriminate patients from those with malignancies of other histological types

**DOI:** 10.1186/s13048-018-0458-0

**Published:** 2018-09-15

**Authors:** Masaki Kobayashi, Kenjiro Sawada, Koji Nakamura, Akihiko Yoshimura, Mayuko Miyamoto, Aasa Shimizu, Kyoso Ishida, Erika Nakatsuka, Michiko Kodama, Kae Hashimoto, Seiji Mabuchi, Tadashi Kimura

**Affiliations:** 1 Departments of Obstetrics and Gynecology, Osaka University Graduate School of Medicine, 2-2 Yamadaoka, Suita, Osaka, 565-0871 Japan; 2 Department of Molecular Oncology, H. Lee Moffitt Cancer Center & Research Institute, Tampa, FL USA

**Keywords:** microRNA, miR-1290, Ovarian cancer, HGSOC, Biomarker

## Abstract

**Background:**

microRNAs (miRNAs) stably exist in circulating blood encapsulated in extracellular vesicles such as exosomes; therefore, serum miRNAs have the potential to serve as novel cancer biomarkers. New diagnostic markers to detect high grade serous ovarian cancer (HGSOC) are urgently needed. The aim of this study was to identify miRNAs specific to HGSOC and analyze whether serum miRNA can discriminate HGSOC patients from healthy controls or patients with ovarian malignancies of other histological types.

**Methods:**

Exosomes from ovarian cancer cell lines were collected and exosomal miRNAs extracted. miRNA microarray analysis revealed several elevated miRNAs specific to HGSOC. Among these, we focused on miR-1290. Sera from 70 ovarian cancer patients and 13 healthy controls were gathered and its expression levels detected by quantitative real-time polymerase chain reaction.

**Results:**

In HGSOC patients, serum miR-1290 was significantly overexpressed compared to in healthy controls (3.52 fold; *P* = 0.03), unlike in patients with ovarian cancers of other histological types. The relative expression of miR-1290 was higher in advanced stages of HGSOC than in early stages (4.23 vs. 1.58; *P* = 0.23). Its expression significantly decreased after operation (5.87 to 1.17; *P* < 0.01), indicating that this miRNA reflects tumor burden. A receiver operating characteristic curve analysis showed that at the cut-off of 1.20, the sensitivity and specificity were 63% and 85% respectively for discriminating patients with HGSOC (area under the curve [AUC] = 0.71) from healthy controls, and at the cut-off of 1.55, the sensitivity and specificity were 47% and 85% respectively for discriminating patients with HGSOC (AUC = 0.76) from those with malignancies of other histological types.

**Conclusions:**

Serum miR-1290 is significantly elevated in patients with HGSOC and can be used to discriminate these patients from those with malignancies of other histological types; it is a new potential diagnostic biomarker for HGSOC.

## Background

Epithelial ovarian cancer (EOC) is one of the most common gynecologic malignancies and the fifth leading cause of cancer-related death in women [1]. The most common histological subtype of EOC is high grade serous ovarian carcinoma (HGSOC). HGSOC is asymptomatic in the early stages and causes rapid dissemination through the peritoneum; therefore, it is typically diagnosed at advanced stages when it is incurable [2]. The standard treatment for HGSOC is debulking surgery, followed by repeated courses of platinum- and taxane-based chemotherapy. However, most patients with HGSOC eventually have a relapse despite receiving intensive treatments with a 5-year survival rate of patients with advanced stage being below 30%. In contrast, the few patients who are diagnosed at stage I have a 5-year survival rate of over 90% [3], indicating that the early detection of ovarian cancer can drastically improve the prognosis of patients [4].

A feasible way to diagnose cancer at an early stage is to establish a useful and comprehensive diagnostic biomarker. To date, only 2 biomarkers, cancer antigen 125 (CA125) and human epididymis 4 (HE4) have been approved by the Food and Drug Administration as biomarkers for EOC [5]. Several studies have reported that HE4 has the highest sensitivity for detecting EOC as a single biomarker; while a combined biomarker of CA125 and HE4 is a more accurate predictor of malignancy than either alone [6–8]. HE4 has not been widely used in clinical settings despite its high sensitivity, and conventional CA125 remains the most widely used diagnostic serum biomarker among patients with EOC [7]. Therefore, pelvic examination, transvaginal ultrasonography, and CA125 tests are performed during routine diagnostic procedures; however, they have failed to detect the disease at an early stage and thus have not significantly reduced the mortality rate of patients with EOC [9]. It is obvious that there is an urgent need to develop a new biomarker for HGSOC to detect it at earlier stages and to provide better treatment options.

Emerging evidence has revealed posttranscriptional regulation of gene expression by microRNAs (miRNAs). miRNAs consist of small non-coding RNAs of approximately 22 nucleotides. Aberrant expression of miRNAs has been identified as a key factor in cancer tumorigenesis and progression. miRNAs are found in the cell-free fraction of blood and can be reliably measured [10] since miRNAs circulating in the serum are present in a variety of forms, such as within small extracellular microvesicles known as exosomes where they are protected from endogenous RNase degradation [11]. Therefore, they have the potential to serve as a disease-specific biomarker for EOC. While various studies have suggested the clinical relevance of circulating miRNAs as diagnostic and prognostic biomarkers for EOC, robust studies evaluating circulatory exosomal miRNA signatures in HGSOC have yet to be reported [12].

With these in mind, we collected exosomes from the culture media of serous EOC cell lines, performed a miRNA microarray, and found that miR-1290 was specifically elevated in these EOC-derived exosomes. To evaluate the potential of miR-1290 as a biomarker for EOC (specifically HGSOC), its expression level in patients was analyzed and compared with that in healthy controls or in patients with malignancies of other histological types.

## Methods

### Materials

Dulbecco’s modified Eagle medium and Roswell Park Memorial Institute 1640 medium were obtained from Nacalai Tesque (Kyoto, Japan). Fetal bovine serum (FBS; #172012) was purchased from Sigma Aldrich (St. Louis, MO). Antibody against CD63 (#11–343-C025) was purchased from EXBIO (Vestec, Czech Republic). Donkey anti-mouse IgG 10 nm gold (#ab39593) was purchased from Abcam (Cambridge, UK).

### Cell culture

TYK-nu cell line was purchased from Health Science Research Resources Bank (Osaka, Japan). HeyA8 cell line was generously contributed by Dr. Anil Sood (MD Anderson Cancer Center, Houston, TX). IOSE cell line was generously contributed by Dr. Masaki Mandai (Kyoto University, Kyoto, Japan). Briefly, ovarian surface epithelial cells were collected from normal ovary and transfected with SV40 large T antigen and human telomerase reverse transcriptase [13]. These cell lines were cultured in optimal medium according to the suppliers’ recommendations.

### Exosome isolation and miRNA extraction from exosomes

Exosomes were isolated from conditioned medium (CM) by an ultracentrifugation method as previously described [14]. The exosome-containing pellet was resuspended in phosphate-buffered saline (PBS) and the amount of exosomal protein was assessed by the Lowry method (Bio-Rad, Hercules, CA). Total RNA was extracted using TRIzol reagent (Life Technologies, Carlsbad, CA: #15596–018).

### Electron microscopy

The morphology and characteristics of exosomes were verified by transmission electron microscopy using a H-7650 transmission electron microscope from Hitachi (Tokyo, Japan) as described [15].

### Exosomal miRNA microarray

A miRNA microarray using the GeneChip miRNA 4.0 array (Affymetrix, Santa Clara, CA) was performed and analyzed by Filgen (Nagoya, Japan). Briefly, 1000 ng of each miRNA sample was biotin-labeled using a Flash TagTM Biotin HSR RNA labeling kit for Affymetrix GeneChip miRNA arrays (Affymetrix) according to the manufacturer’s protocol. Hybridization solution was prepared using 110.5 μL hybridization master mix and 21.5 μL biotin-labeled sample. The array was incubated using a GeneChip Hybridization Oven 645 (Affymetrix) and washed using a GeneChip Fluidics station 450 (Affymetrix) according to the manufacturer’s protocol. The washed array was analyzed using a GeneChip Scanner 3000 7G (Affymetrix).

### Patients and samples

In a retrospective cohort study, data were reviewed from patients with ovarian cancer treated at Osaka University Hospital (Suita, Osaka) between January 2013 and May 2015. The study was approved by the Institutional Review Board of the institute and conformed to the provisions of the Declaration of Helsinki. Written informed consent was obtained from every participant for the use of their samples.

Blood samples were collected from healthy volunteers (*n* = 13) and patients with ovarian cancer (*n* = 70). From 16 HGSOC patients, blood samples were collected twice at the timing of admission for primary debulking surgery (PDS) (2 or 3 days before surgery) and the initial postoperative chemotherapy (approximately 28 days after surgery). The samples were centrifuged at 1500 x g for 10 min at 4 °C and the upper sera phases were stored in aliquots at − 80 °C until use.

### miRNA extraction from serum

miRNAs were extracted from 200 μL serum samples using an miRNeasy Serum/Plasma Kit (QIAGEN, Hilden, Germany) according to the manufacturer’s protocol. The eluates containing miRNAs were stored at − 80 °C until use.

### Quantitative real-time polymerase chain reaction (qRT-PCR) of miR-1290

The expression of miR-1290 was evaluated by quantitative real-time polymerase chain reaction (qRT-PCR). TaqMan miRNA assays (#4366596, Applied Biosystems, Foster City, CA) with specific reverse transcription primers and probes were used to quantify the expression of mature human miR-1290 (5′ – UGGAUUUUUGGAUCAGGGA – 3′). Before RNA extraction, 100 fmol/mL of synthesized cel-miR-39 (#4427975, Applied Biosystems) was added to an equal volume of serum to serve as a normalizer. The relative levels of miR-1290 in serum were expressed using the 2^−ΔCt^ method, in which ΔCt = Ct^miR-1290^ − Ct^cel-miR-39^. qRT-PCR was performed in triplicate with the StepOnePlus Real-Time PCR system (Applied Biosystems).

### Statistical analysis

JMP software version 13.0.0 (SAS Institute, Tokyo, Japan) was used for statistical analysis and the construction of statistical plots. Differences in miRNA expression levels in non-paired samples were tested by the Mann-Whitney U test. The Wilcoxon test was used to compare miRNA expression in paired samples. The values are presented as the mean ± SD and a two-sided *P* < 0.05 was considered to indicate a statistically significant difference. In the graphs showing the relative expression of miR-1290 in the patients’ sera, Diagnostic performance for each biomarker individually and in combination was evaluated using receiver operating characteristic (ROC) curve analysis and by reporting the area under the curve (AUC).

## Results

### The identification of candidate miRNAs

**Fig. 1:**
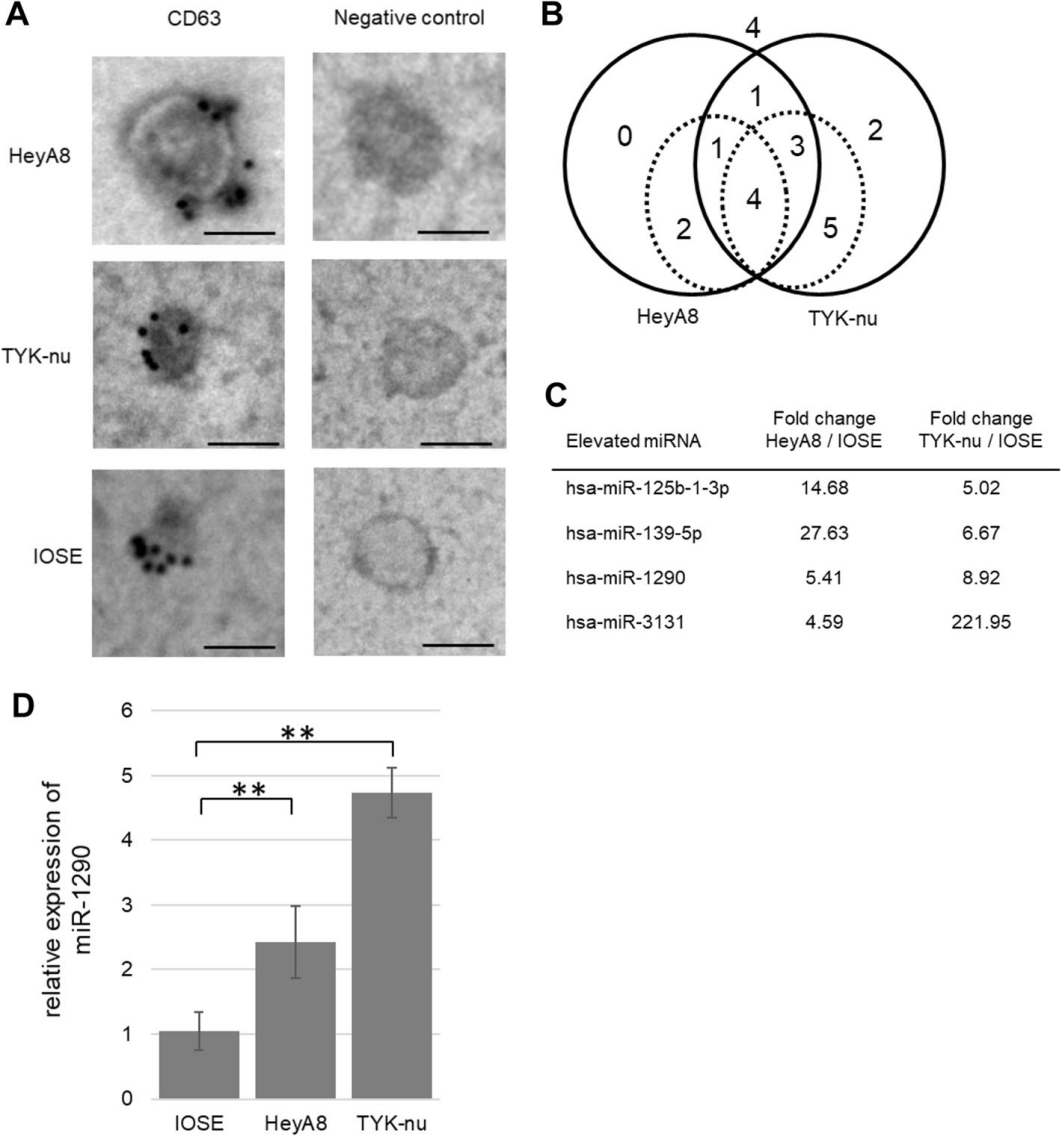
The collection of exosomes from EOC cell lines and the analyses of exosomal miRNAs. (**a**) Electron microscopy. Exosomes were immunogold-labeled with anti-CD63 antibody and transmission electron micrographs of isolated exosomes secreted from 2 HGSOC cell lines (HeyA8, upper panel;TYK-nu, middle panel) are shown. Immortalized ovarian surface epithelium (IOSE) cells (bottom panel) were used as a non-malignant control. Scale bar, 100 nm. (**b**) Venn diagrams showing the relative miRNAs expressed in HeyA8 and TYK-nu vs. IOSEs by exosomal miRNA microarray. Circles with solid lines represent counts of exosomal miRNAs with expression levels increased by > 3.0-fold. Circles with dotted lines represent counts of miRNAs with expression levels increased by > 4.0-fold. (**c**) Summary of exosomal miRNA microarray. Exosomal miRNAs that were upregulated by more than 4.0-fold in both HeyA8 and TYK-nu vs. IOSEs are listed. (**d**) qRT-PCR of miR-1290. The relative expression of miR-1290 in HGSOC-derived exosomes is shown. IOSE-derived exosomes were used as a control. Data are the mean ± standard deviation (SD) of 3 experiments. **, *P* < 0.01

First, by multi-step ultracentrifugation, exosomes were collected from the CM of HeyA8, TYK-nu and IOSE. HeyA8 and TYK-nu were derived from HGSOC cells and IOSE is an immortalized normal ovarian epithelial cell line used as a benign control. Exosomes exhibited a round-shaped morphology, showed positive staining for CD63, which is a representative exosomal marker, and were approximately 100 nm in size as determined by electron microscopy (Fig. 1a) [16]. miRNA microarray analysis revealed the expression profiles of miRNAs which were highly expressed in exosomes derived from the HGSOC cells compared with those from the IOSE cells (Fig. 1b). Among those, 4 miRNAs (miR-129b-1-3p, miR-139-5p, miR-1290, and miR-3131) were identified as highly expressed (> 4.0) in both HeyA8- and TYK-nu-derived exosomes (Fig. 1c). miRNA qRT-PCR assay validated that the expression level of miR-1290 was higher by 5.41-fold in HeyA8 exosomes and 8.92-fold in TYK-nu exosomes compared with that in IOSE exosomes (Fig. 1d).

### Serum miRNA-1290 expression levels in EOC

Since the expression of miR-1290 was highly expressed in exosomes derived from HGSOC cell lines, we were encouraged to determine miR-1290 expression levels in the sera of patients with EOC and to evaluate its diagnostic value as a biomarker. A total of 83 sera samples were collected from healthy controls (*n* = 13) and patients with EOC (*n* = 70), and miR-1290 expression levels were examined by qRT-PCR. Clinical characteristics of patients and healthy controls are presented in Table 1. Out of 70 EOC patients, 30 (43%) had serous ovarian cancer, all of which were diagnosed as HGSOC. Among these, 22 (72%) had advanced stage (stage III/IV) disease and 8 (27%) had early stage (stage I/II) disease (Table 2).

**Table 1:** Characteristics of the participants

	Healthy controls (*n* = 13)	Epithelial ovarian cancer (*n* = 70)	
Age, years Median (range)	34 (23–45)	58 (33–78)	
Histological type		Serous	30 (43%)
		Clear-cell	18 (26%)
		Endometrioid	12 (17%)
		Mucinous	10 (14%)
FIGO Stage		I	31 (44%)
		II	6 (9%)
		III	31 (44%)
		IV	2 (3%)

Abbreviations: *FIGO* Federation of Gynecology and Obstetrics

**Table 2:** FIGO stages of the HGSOC patients

	HGSOC patients (*n* = 30)	
FIGO Stage	I	5 (17%)
	II	3 (10%)
	III	21 (70%)
	IV	1 (3%)

Abbreviations: *FIGO* Federation of Gynecology and Obstetrics, *HGSOC* high grade serous ovarian cancer

**Fig. 2:**
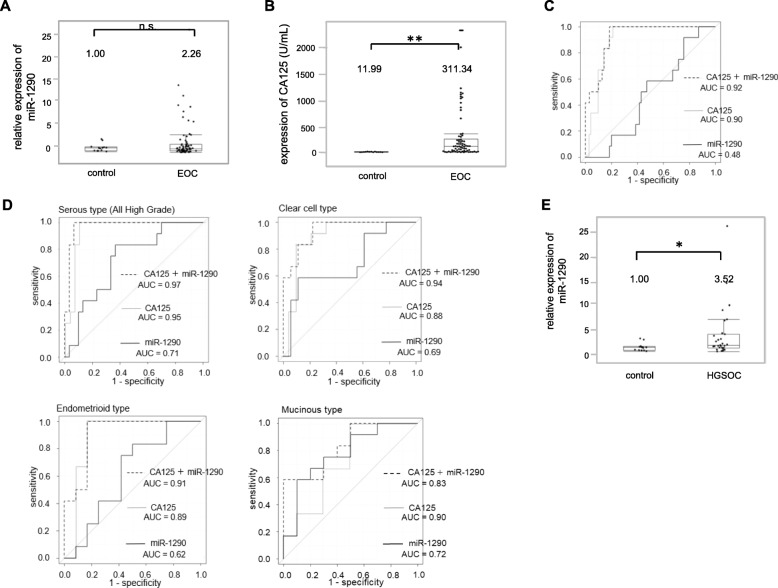
Serum miR-1290 expression is significantly elevated in HGSOC patients. (**a**) Relative miR-1290 expression levels in sera from healthy controls (*n* = 13) and EOC patients (*n* = 70) are shown. The average miR-1290 expression level in healthy controls was normalized to 1.0. The box-and-whisker plots indicate the median and interquartile range. (**b**) CA125 values in sera from healthy controls (*n* = 13) and EOC patients (*n* = 70) are shown. The box-and-whisker plots indicate the median and interquartile range. (**c**) ROC curves for the identification of patients with EOC (*n* = 70) vs. healthy controls (*n* = 13) based on the expression of CA125 (gray line), miR-1290 (black line), and the combination of both (dotted line). The AUC values are shown on the graphs. (**d**) ROC curves for the identification of patients with ovarian cancer of each histological subtype (serous: *n* = 30, clear-cell: *n* = 18, endometrioid: *n* = 12, mucinous: *n* = 10) vs. healthy controls (*n* = 13). (**e**) Relative miR-1290 expression levels in sera from healthy controls (*n* = 13) and HGSOC patients (*n* = 30) are shown. **, *P* < 0.01; *, *P* < 0.05; n.s., not significant

The relative expression level of miR-1290 in EOC patients was 2.26 compared to that in healthy controls (Fig. 2a); however, there was no significant difference between the 2 groups (*P* = 0.89). In contrast, CA125 value in EOC patients was 311.34 U/mL and significantly elevated comapred to that in healthy controls (11.99 U/mL) (*P* < 0.01; Fig. 2b). The diagnostic performance value of serum miR-1290 was calculated by ROC curve analysis to discriminate EOC patients from healthy controls. When the cut-off of miR-1290 was set at 1.49, the sensitivity and specificity were 0.51 and 0.57, respectively (AUC = 0.48; Fig. 2c). For comparison, a ROC curve to discriminate EOC patients from healthy controls was similarly calculated for CA125. When the cut-off of CA125 was set at 76 U/mL, the sensitivity and specificity were 1.00 and 0.61, respectively (AUC = 0.90). Thus, CA125 served as a better biomarker to disciminate all EOC patients from healthy controls. The combination of the expression levels of miR-1290 and CA125 for ROC curve analysis did not significantly improve the AUC value (from 0.90 to 0.92; *P* = 0.08; Fig. 2c), indicating that this model failed to distinguish EOC patients from healthy controls. EOC consists of the following 4 major histological subtypes: serous (in our case series, all such cases were diagnosed as HGSOC), clear-cell, endometrioid, and mucinous. Using a cut-off of 1.20, the miR-1290 classification model had AUC, sensitivity, and specificity values of 0.71, 0.63, and 0.85, respectively, for HGSOC; 0.69, 0.58, and 0.89, respectively, for clear-cell EOC; 0.62, 0.50, and 0.83, respectively, for endometrioid EOC; and 0.78, 0.58, and 0.90, respectively, for mucinous EOC (Fig. 2d). Among these, the diagnostic performance of HGSOC was good and the combination of miR-1290 and CA125 significantly improved AUC value from 0.71 to 0.97 (*P* < 0.01; Fig. 2d). Indeed, the relative miR-1290 expression in HGSOC patients was 3.52 and significantly higher than that in healthy controls (*P* = 0.03; Fig. 2e). Collectively, our data suggested that serum miR-1290 might be a useful biomarker to detect HGSOC in patients.

### Serum miR-1290 is significantly elevated in HGSOC and reflects tumor burden

**Fig. 3:**
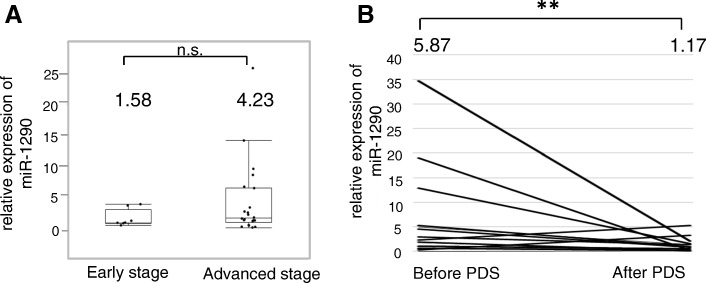
Serum miR-1290 expression reflects HGSOC tumor burden. (**a**) Relative serum miR-1290 expression levels between HGSOC patients at early (I + II) (*n* = 8) and advanced (III + IV) stage (*n* = 22) are shown. The average miR-1290 expression level in healthy controls (*n* = 13) was normalized to 1.0. The box-and-whisker plots indicate the median and interquartile range. (**b**) Relative miR-1290 expression levels in matched serum samples from HGSOC patients before and after primary debulking surgery (PDS). Individual changes in the serum expression of miR-1290 in HGSOC patients before and after PDS are shown. The average miR-1290 expression level in healthy controls (*n* = 13) was normalized to 1.0. **, *P* < 0.01; n.s., not significant

While the relative expression of miR-1290 in stage I-II EOC patients was 1.58, that in stage III-IV patients was increased to 4.23 (*P* = 0.23; Fig. 3a). In order to find out whether miR-1290 reflected tumor burden, sera were collected from 16 HGSOC patients twice just before PDS and approximately 28 days after PDS as well as before the following chemotherapy, and its expression level was compared (Fig. 3b). While the relative expression level before PDS was 5.87, that after PDS significantly declined to 1.17 (*P* < 0.01), suggesting that serum miR-1290 reflects tumor burden and implying that it is derived from HGSOC cells.

### Serum miR-1290 has the potential to differentiate HGSOC patients from non-HGSOC patients

**Fig. 4:**
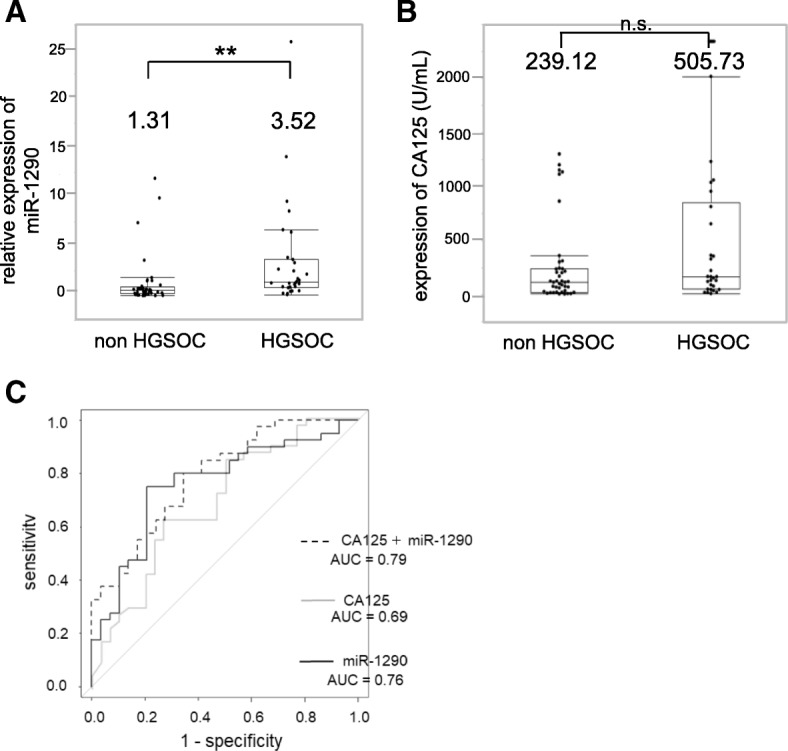
Diagnostic performance of miR-1290 for the discrimination of HGSOC from non-HGSOC. (**a**) Relative miR-1290 expression levels in sera from HGSOC patients (*n* = 30) and patients with EOC other than HGSOC (*n* = 40). The average miR-1290 expression level in healthy controls (*n* = 13) was normalized to 1.0. The box-and-whisker plots indicate the median and interquartile range. (**b**) CA125 expression levels in sera from HGSOC patients (*n* = 30) and patients with EOC other than HGSOC (*n* = 40). The box-and-whisker plots indicate the median and interquartile range. (**c**) ROC curves for the identification of HGSOC patients from patients with EOC other than HGSOC (*n* = 40) based on the expression of CA125 (gray line), miR-1290 (black line), and the combination of both (dotted line). **, *P* < 0.01; n.s., not significant

Several phase III studies have shown that interval debulking surgery following neoadjuvant chemotherapy (NAC) is less invasive with non-inferior clinical outcomes compared to PDS followed by chemotherapy for treating advanced stage ovarian cancer [17–19]. However, Tian et al. showed that platinum-based first-line chemotherapy was much less effective for mucinous and clear-cell types, although it is effective for serous and endometrioid types [20]. Therefore, it would be clinically beneficial if we could establish an appropriate way to determine which patients should be treat with NAC. For this reason, it is meaningful to find a biomarker specific to HGSOC which is able to discriminate it from EOC of other histological types. Among 70 sera samples from EOC patients, the expression level of miR-1290 was compared between HGSOC patients (*n* = 30) and patients with EOC other than HGSOC (*n* = 40). While the expression level of miR-1290 in patients with EOC other than HGSOC was 1.32, that in HGSOC patients was 3.52, which was significantly higher (*P* < 0.01, Fig. 4a). In contrast, while CA125 value in patients with EOC other than HGSOC was 239.12 U/mL, that in HGSOC patients was 505.73 U/mL and no significant differences were seen (*P* = 0.08; Fig. 4b). When the cut-off of the relative miR-1290 expression was set at 1.55, the sensitivity was 0.47 and the specificity was 0.85 (AUC = 0.76; Fig. 4c). For comparison with CA125, ROC curve analysis for discriminating patients with HGSOC from those with EOC other than HGSOC was similarly calculated. The sensitivity and specificity of serum CA125 were 0.73 and 0.63, respectively (AUC = 0.69). Although there was no significant difference between the AUC values of miR-1290 and CA125, the combined AUC value was 0.79 and improved from the AUC values of either alone.

## Discussion

Recently, aberrant expression of various miRNAs has been identified to be closely associated with the initiation and progression of cancer [10, 21, 22]. In this study, through a comprehensive study with miRNA microarray, we identified miR-1290 as being significantly elevated in HGSOC-derived exosomes. Furthermore, we found that serum miR-1290 was significantly elevated in patients with HGSOC and could discriminate such patients from those with malignancies of other histological types, suggesting its potential as a novel diagnostic biomarker for HGSOC. Although CA125, a conventional biomarker of EOC, showed a better performance to discriminate all EOC patients from healthy controls than serum miR-1290, miR-1290 showed a better performance to discriminate HGSOC patients from non-HGSOC patients than CA125.

Our data suggests that miR-1290 reflects tumor burden and is derived from HGSOC cells. So far, studies regarding the role of miR-1290 in cancer biology have been limited. Li et al. showed that miR-1290 was elevated in the serum of patients with low-stage pancreatic cancer and could be a highly sensitive and specific biomarker for pancreatic cancer [23]. Wu et al. indicated that miR-1290 was highly expressed in clinical colon cancer tissue and that it could impair cytokinesis and affect the reprogramming of colon cancer cells [24]. However, to the best of our knowledge, the role of miR-1290 in EOC has not been reported. Bioinformatics analysis using the miRTargetLink human database [25] suggested several putative target genes of miR-1290 (Table 3). One of the predictive targets was melastatin-related transient receptor potential cation channel, subfamily M, member 7 (TRPM7), which plays a role in the inhibition of proliferation, colony formation, migration, and invasion in ovarian cancer cell lines [26]. Another predictive target gene was C8orf4 or N-myc downstream regulated gene 1 (NDRG1). Xu et al. reported that high expression of C8orf4 was significantly correlated with poor differentiation in ovarian cancers [27]. NDRG1 overexpression decreased adhesion, proliferation and apoptosis, and induced G0/G1 cell cycle arrest in ovarian cancer cells; expression of p53 and p21 was also increased [28]. Further studies would be indispensable for the identification of the true target genes of miR-1290 in HGSOC to clarify its role.

**Table 3:**
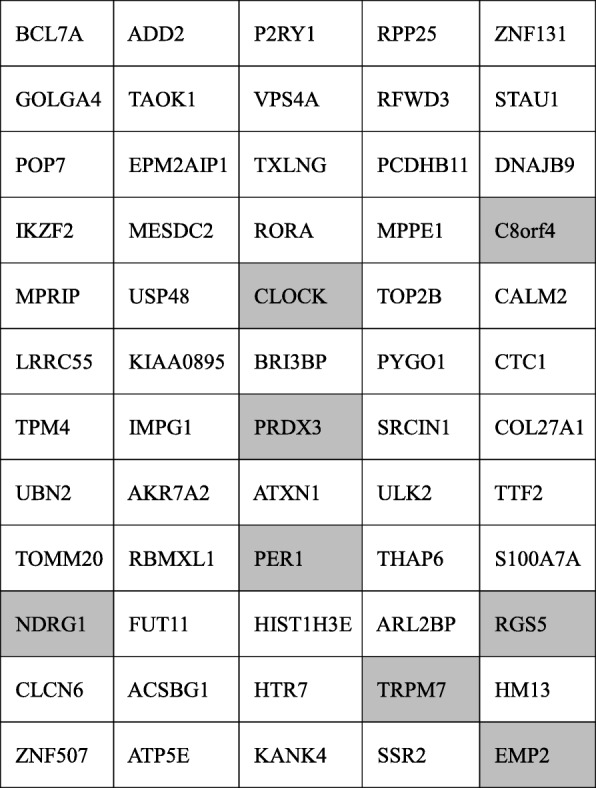
Putative targeted genes of miR-1290 as suggested by the miRTargetLink human database (https://ccb-web.cs.uni-saarland.de/mirtargetlink/)

Among the 60 genes which were considered to be interacting with miR-1290, those in the gray cells have been reported to be correlated with ovarian cancer

Exosomes are cell-derived extracellular vesicles that promote cell-cell communication, shuttling various molecules including miRNAs to recipient cells [29]. In the blood, most miRNAs are present in exosomes as exosomal miRNAs and are not degraded by proteases [29]; therefore, exosomal miRNAs in body fluids have the potential to diagnose normal or abnormal processes or diseases [30]. Several studies have shown the clinical relevance of circulating miRNAs as diagnostic and prognostic biomarkers for ovarian cancer, using blood plasma or serum as we reviewed previously [12]. In 2008, Taylor et al. first reported that 8 exosomal miRNAs (miR-21, miR-141, miR-200a, miR-200b, miR-200c, miR-203, miR-205, and miR-214) from sera were elevated in ovarian cancer patients as compared to benign controls. These miRNA signatures from exosomes were parallel to those from the originating tumor cells, indicating that circulating miRNA profiles accurately reflect the tumor profiles [31]. Recently, Yokoi et al. developed a novel predictive model using a combination of 8 circulating serum miRNAs (miR-200a-3p, miR-766-3p, miR-26a-5p, miR-142-3p, let-7d-5p, miR-328-3p, miR-130b-3p, and miR-374a-5p) which could distinguish EOC patients from healthy controls with high sensitivity and specificity (0.92 and 0.91, respectively) [32]. They demonstrated that most of these miRNAs were packaged in extracellular vesicles, including exosomes, and were derived from ovarian cancer cells. In a cohort of 56 HGSOC patients, Shah et al. reported a combination of miR-375 and CA-125 was the strongest discriminator of healthy versus HGSOC serum with an AUC of 0.956 and the combination of miR-34a-5p and CA-125 the strongest predictor of completeness of surgical resection with an AUC of 0.818 [33]. However, despite these interesting reports, progress for the development of reliable serum biomarkers for the early detection of HGSOC remains very limited due to the small size of available research cohorts.

Given that HGSOC is the most common EOC and responds well to chemotherapy, if we had a biomarker to differentiate HGSOC from non-HGSOC, it would be clinically advisable to choose NAC or PDS for the treatment of stage III/VI ovarian cancer. Herein, we showed that miR-1290 is better than CA125 for distinguishing HGSOC from non-HGSOC, suggesting the potential of this miRNA as a biomarker. Thus, in addition to CA125 and HE4, the measurement of miR-1290 may provide a more accurate diagnosis for EOC.

Several limitations should be acknowledged. The sample size is too small to reach a solid conclusion. Large and prospective registry-embedded trials would be needed to strengthen our hypothesis that serum miR-1290 can serve as a biomarker of HGSOC. Besides, the mechanisms behind why miR-1290 is up-regulated in HGSOC serum need to be explored. Further studies would be indispensable for the identification of putative target genes of miR-1290 in HGSOC to clarify its role. Endogenous controls for miRNA normalization remain critical for the reliable quantification of miRNAs. RNU-6B, RNU-48, or miR-16 are commonly used as endogenous controls; however, no definitive control gene has been established [12]. In this study, exogenous miRNA (synthesized cel-miR-39) was used as a control for miRNA normalization. The establishment of an appropriate normalization method is indispensable for clinical use.

## Conclusions

Serum miR-1290 reflects tumor burden and shows promise as a potential biomarker to aid in the diagnosis of HGSOC. Elevated serum miR-1290 has the potential to distinguish HGSOC patients from non-HGSOC patients. The addition of this miRNA measurement to conventional biomarkers such as CA125 and HE4 may provide an accurate diagnosis for EOC. Further studies would be needed to elucidate the molecular mechanisms of miR-1290 in HGSOC tumorigenesis and progression.

## Abbreviations

AUC: Area under the curve
CA125: Cancer antigen 125
CM: Conditioned medium
EOC: Epithelial ovarian cancer
FIGO: Federation of Gynecology and Obstetrics
HE4: Human epididymis 4
HGSOC: High grade serous ovarian carcinoma
IOSE: Immortalized ovarian surface epithelium
miRNAs: microRNAs
NAC: Neoadjuvant chemotherapy
NDRG1: N-myc downstream regulated gene 1
PBS: Phosphate-buffered saline
PDS: Primary debulking surgery
qRT-PCR: Quantitative real-time polymerase chain reaction
ROC: Receiver operating characteristics
TRPM7: Transient receptor potential cation channel, subfamily M, member 7
